# Crystal structure of *trans*-di­chlorido­(4-nitro­aniline-κ*N* ^1^)(piperidine-κ*N*)platinum(II)

**DOI:** 10.1107/S2056989015009196

**Published:** 2015-05-20

**Authors:** Chi Nguyen Thi Thanh, Truong Hoang Van, Thong Pham Van, Ngan Nguyen Bich, Luc Van Meervelt

**Affiliations:** a Chemistry Department, Hanoi National University of Education, 136 – Xuan Thuy – Cau Giay, Hanoi, Vietnam; b Chemistry Department, KU Leuven, Celestijnenlaan 200F, B-3001 Leuven (Heverlee), Belgium

**Keywords:** crystal structure, *trans*-platinum(II) complexes, hydrogen bonding

## Abstract

In the title complex, [PtCl_2_(C_5_H_11_N)(C_6_H_6_N_2_O_2_)], the Pt^II^ metal atom displays a slightly distorted *trans*-PtN_2_Cl_2_ square-planar coordination geometry. The dihedral angle between the mean plane of the benzene and piperidine rings is 89.03 (3)°. In the crystal structure, inversion dimers are formed *via* N—H⋯Cl hydrogen-bond inter­actions, resulting in chains parallel to the [001] direction. The benzene rings within the chains show π–π stacking inter­actions [centroid-to-centroid distances of 3.801 (3) Å] and neighbouring chains inter­act *via* N—H⋯O hydrogen bonds.

## Abstract 2

The packing of the title compound features N—H⋯Cl hydrogen bonds and π–π stacking inter­actions, which form one-dimensional chains of mol­ecules parallel to [001] further linked *via* N—H⋯O inter­actions.

## Chemical context   

The title compound is one of many complexes which have been synthesized for the purpose of potential medical applications (Klein & Hambley, 2009 ▸; Wilson & Lippard, 2014 ▸; Peng *et al.*, 2014 ▸). It is notable that according to the procedure used for the synthesis of complexes of the type *cis*-[PtCl_2_(piperidine)(another amine)] (piperidine hereafter denoted Pip) (Dinh & Da, 2003 ▸; Nguyen Thi Thanh *et al.*, 2014 ▸), the reaction between K[PtCl_3_(Pip)] and *p*-nitro­aniline under appropriate conditions gave no *cis* complex, as expected, but instead gave the *trans*-[PtCl_2_(*p*-nitro­aniline)(Pip)] derivative, (I).
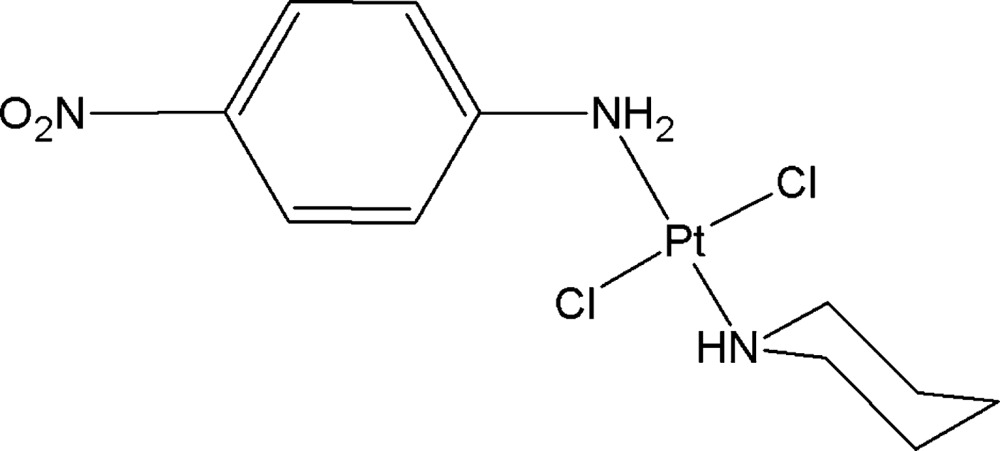


To explain this we suppose that *p*-nitro­aniline first coordinates with Pt^II^ *via* the N atom of the amino group to form *cis*-[PtCl_2_(*p*-nitro­aniline)(Pip)] based on the *trans* effect. Then, in the reaction solution, the *cis* complex converts into the *trans* complex and the thermodynamics of this conversion are currently under investigation by us.

The anti­cancer activity of the title compound was tested according to the method described by Skehan *et al.* (1990 ▸) against four human cancer cell lines (HepG2, RD, MCF7 and Fl). The IC_50_ values calculated based on OD values taken on an Elisa instrument at 515–540 nm are >10, 4.86, >10 and 8.25 µg ml^−1^, respectively.

## Structural commentary   

**Figure 1:**
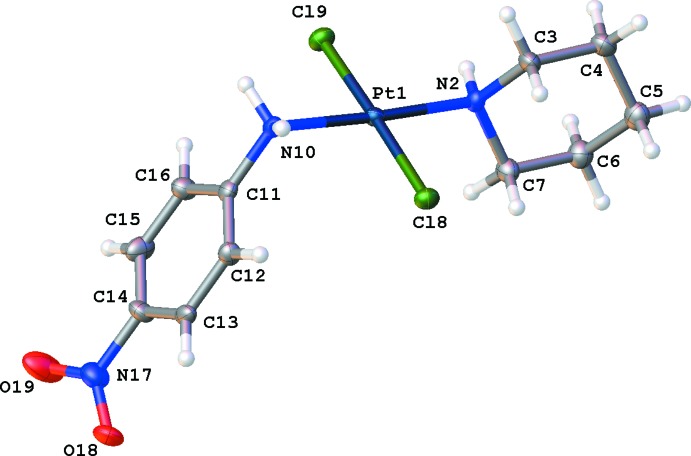
The mol­ecular structure of the title compound, with displacement ellipsoids drawn at the 50% probability level.

The mol­ecular structure of the title compound is illustrated in Fig. 1 ▸ and surprisingly shows a *trans* arrangement of the two Cl atoms [Cl8—Pt1—Cl9 = 177.84 (4)°]. The piperidine ring adopts the usual chair conformation, with the N2—Pt1 bond in the equatorial position. The piperidine ring is oriented nearly perpendicular to the coordination plane of the Pt^II^ atom, thereby reducing the van der Waals repulsion; the dihedral angle between the least-squares mean planes through the piperdine ring and the four atoms coordinated to the Pt atom is 89.6 (2)°. One short intra­molecular contact is observed, *i.e.* H7*B*⋯Cl8 = 2.83 Å. The mean planes through the piperidine ring and the benzene ring make a dihedral angle of 89.0 (3)°. The dihedral angle between the mean planes of the nitro substituent and the benzene ring is 16.6 (3)°.

## Supra­molecular features   

**Figure 2:**
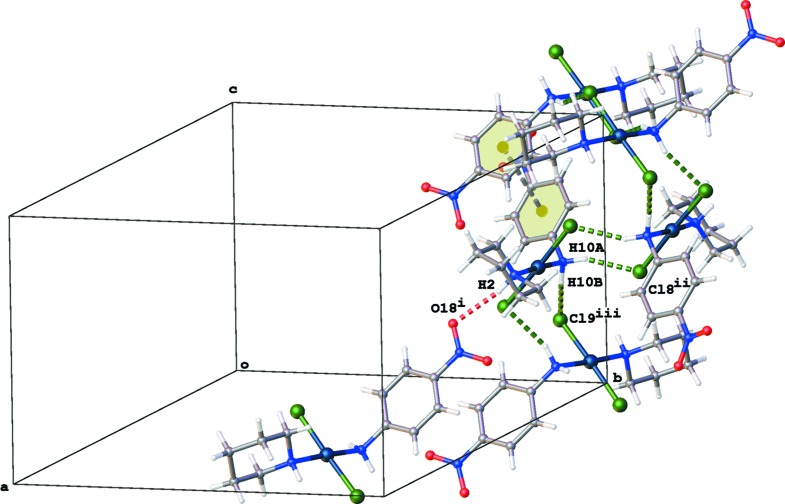
Partial packing diagram of the title compound, showing a chain of mol­ecules formed parallel to the [001] direction *via* N—H⋯Cl inter­actions (green dotted lines) and π–π inter­actions (grey dotted line). Neighbouring chains inter­act *via* N—H⋯O hydrogen bonds (red dotted line).

In the crystal, inversion dimers are formed *via* N—H⋯Cl inter­actions between the aniline N atom and both Cl atoms, resulting in chains of mol­ecules along the [001] direction (Fig. 2 ▸ and Table 1 ▸). Within these chains, π–π inter­actions occur between the aromatic rings [*Cg*⋯*Cg* ^iv^ = 3.801 (3) Å; *Cg* is the centroid of the C11–C16 ring; symmetry code: (iv) −*x*, *y*, −*z* + 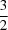; Fig. 2 ▸]. Neighbouring chains are linked *via* N—H⋯O hydrogen bonds between the piperidine N atom and a nitro O atom (Fig. 2 ▸ and Table 1 ▸).

## Database survey   

A search of the Cambridge Structural Database (Version 5.36; last update February 2015; Groom & Allen, 2014 ▸) for Pt complexes with Pt coordinated to exactly two Cl atoms and two N atoms gave 713 hits. The majority of these Pt complexes display a *cis* coordination of the Cl atoms (474 structures), with the remaining 239 structures showing a *trans* coordination. There is no difference in the Pt—Cl distances between both configurations. The average Pt—Cl distances are 2.300 (15) and 2.299 (12) Å for the *cis* and *trans* arrangements, respectively, and correspond to the observed distances of 2.3039 (11) and 2.2917 (12) Å for Pt1—Cl8 and Pt1—Cl9, respectively.

## Synthesis and crystallization   

The starting complex K[PtCl_3_(piperidine)] (0.425 g, 1 mmol), prepared according to the synthetic procedure of Da *et al.* (2001 ▸) with slight modifications, was dissolved in water (10 ml) and filtered to afford a clear solution. To this solution, *p*-nitro­aniline (1 mmol) in ethanol (10 ml) was added gradually while stirring at 413–318 K. After 1 h, a brown powder appeared and the reaction mixture was then stirred further for 24 h until all the precipitate was completely dissolved. The solvent was removed *in vacuo* to give a brown–yellow product. The product was washed consecutively with a 0.1 *M* HCl solution (2 × 2 ml), warm water (2 × 2 ml) and diethyl ether (2 × 2 ml). The yield was 80%. Single crystals suitable for X-ray determination were obtained by slow evaporation within 12 h from an acetone solution at room temperature. IR (KBr, cm^−1^): 3199, 3113 (ν_NH_); 3070, 2927, 2862 (ν_CH_); 1596, 1525, 1479 (ν_C=C arom_); 1342, 1325 (ν_NO_); ^1^H NMR (CDCl_3_, 500 MHz): δ 8.21 (2H, *d*, ^3^ *J* = 9.0 Hz, Ar-*H*), 7.47 (2H, *d*, ^3^ *J* = 9.0 Hz, Ar-*H*), 5.49 (2H, *br*, O_2_NC_6_H_4_N*H_2_*), 3.66 (1H, *br*, C_5_H_10_N*H*), 3.26 (2H_α_^e^, *d*, ^2^ *J*_ae_ = 13.0 Hz, C_5_ *H_10_*NH), 2.99 (2H_α_^a^, *q*, ^2^ *J*_ae_, ^3^ *J*_aa_, ^3^ *J*_aa(NH)_ = 13.0 Hz, C_5_ *H_10_*NH), 1.69–1.43 (4H_β_, 2H_γ_, *ov*, C_5_ *H_10_*NH). ^13^C{^1^H} NMR (125 MHz, CDCl_3_): δ 149.6, 125.1, 124.2 (O_2_N*C_6_*H_4_NH_2_), 54.0, 27.2, 24.3 (*C_5_*H_10_NH).

## Refinement   

Crystal data, data collection and structure refinement details are summarized in Table 2 ▸. All H atoms were placed at idealized positions and refined in riding mode, with *U*_iso_(H) values assigned as 1.2*U*_eq_ of the parent atoms, with C—H distances of 0.95 (aromatic) and 0.99 Å (methyl­ene), and N—H distances of 0.93 (NH) and 0.92 Å (NH_2_).

## Supplementary Material

Crystal structure: contains datablock(s) I. DOI: 10.1107/S2056989015009196/rz5159sup1.cif

Structure factors: contains datablock(s) I. DOI: 10.1107/S2056989015009196/rz5159Isup2.hkl

CCDC reference: 1400786

Additional supporting information:  crystallographic information; 3D view; checkCIF report

## Figures and Tables

**Table 1:** Hydrogen-bond geometry (, )

*D*H*A*	*D*H	H*A*	*D* *A*	*D*H*A*
N2H2O18^i^	0.93	2.27	3.182(6)	165
N10H10*A*Cl8^ii^	0.92	2.32	3.198(4)	158
N10H10*B*Cl9^iii^	0.92	2.37	3.255(4)	161

Symmetry codes: (i) 
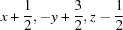
; (ii) 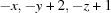; (iii) 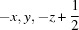.

**Table 2:** Experimental details

Crystal data	
Chemical formula	[PtCl_2_(C_5_H_11_N)(C_6_H_6_N_2_O_2_)]
*M*_r_	489.27
Crystal system, space group	Monoclinic, *C*2/*c*
Temperature (K)	100
*a*, *b*, *c* ()	15.8763(11), 18.5394(11), 10.8707(6)
()	103.119(7)
*V* (^3^)	3116.1(3)
*Z*	8
Radiation type	Mo *K*
(mm^1^)	9.35
Crystal size (mm)	0.35 0.15 0.1

Data collection	
Diffractometer	Agilent SuperNova (single source at offset, Eos detector)
Absorption correction	Multi-scan (*CrysAlis PRO*; Agilent, 2012 ▸)
*T*_min_, *T*_max_	0.538, 1.000
No. of measured, independent and observed [*I* > 2(*I*)] reflections	8156, 3109, 2713
*R*_int_	0.044
(sin /)_max_ (^1^)	0.625

Refinement	
*R*[*F* ^2^ > 2(*F* ^2^)], *wR*(*F* ^2^), *S*	0.032, 0.072, 1.10
No. of reflections	3109
No. of parameters	172
H-atom treatment	H-atom parameters constrained
_max_, _min_ (e ^3^)	2.75, 1.82

Computer programs: *CrysAlis PRO* (Agilent, 2012 ▸), *SHELXS97* and *SHELXL97* (Sheldrick, 2008 ▸) and *OLEX2* (Dolomanov *et al.*, 2009 ▸).

## supplementary crystallographic information

### Crystal data

**Table:**

[PtCl_2_(C_5_H_11_N)(C_6_H_6_N_2_O_2_)]	*F*(000) = 1856
*M**_r_* = 489.27	*D*_x_ = 2.086 Mg m^−^^3^
Monoclinic, *C*2/*c*	Mo *K*α radiation, λ = 0.71073 Å
*a* = 15.8763 (11) Å	Cell parameters from 3549 reflections
*b* = 18.5394 (11) Å	θ = 3.4–28.8°
*c* = 10.8707 (6) Å	µ = 9.35 mm^−^^1^
β = 103.119 (7)°	*T* = 100 K
*V* = 3116.1 (3) Å^3^	, brown
*Z* = 8	0.35 × 0.15 × 0.1 mm

### Data collection

**Table:**

Agilent SuperNova (single source at offset, Eos detector) diffractometer	3109 independent reflections
Radiation source: SuperNova (Mo) X-ray Source	2713 reflections with *I* > 2σ(*I*)
Mirror monochromator	*R*_int_ = 0.044
Detector resolution: 15.9631 pixels mm^-1^	θ_max_ = 26.4°, θ_min_ = 2.8°
ω scans	*h* = −19→15
Absorption correction: multi-scan (*CrysAlis PRO*; Agilent, 2012)	*k* = −17→23
*T*_min_ = 0.538, *T*_max_ = 1.000	*l* = −13→13
8156 measured reflections	

### Refinement

**Table:**

Refinement on *F*^2^	Primary atom site location: structure-invariant direct methods
Least-squares matrix: full	Secondary atom site location: difference Fourier map
*R*[*F*^2^ > 2σ(*F*^2^)] = 0.032	Hydrogen site location: inferred from neighbouring sites
*wR*(*F*^2^) = 0.072	H-atom parameters constrained
*S* = 1.10	*w* = 1/[σ^2^(*F*_o_^2^) + (0.0296*P*)^2^ + 0.5839*P*] where *P* = (*F*_o_^2^ + 2*F*_c_^2^)/3
3109 reflections	(Δ/σ)_max_ = 0.003
172 parameters	Δρ_max_ = 2.75 e Å^−^^3^
0 restraints	Δρ_min_ = −1.82 e Å^−^^3^

### Special details

**Table:**

Geometry. All e.s.d.'s (except the e.s.d. in the dihedral angle between two l.s. planes) are estimated using the full covariance matrix. The cell e.s.d.'s are taken into account individually in the estimation of e.s.d.'s in distances, angles and torsion angles; correlations between e.s.d.'s in cell parameters are only used when they are defined by crystal symmetry. An approximate (isotropic) treatment of cell e.s.d.'s is used for estimating e.s.d.'s involving l.s. planes.
Refinement. Refinement of *F*^2^ against ALL reflections. The weighted *R*-factor *wR* and goodness of fit *S* are based on *F*^2^, conventional *R*-factors *R* are based on *F*, with *F* set to zero for negative *F*^2^. The threshold expression of *F*^2^ > σ(*F*^2^) is used only for calculating *R*-factors(gt) *etc*. and is not relevant to the choice of reflections for refinement. *R*-factors based on *F*^2^ are statistically about twice as large as those based on *F*, and *R*- factors based on ALL data will be even larger.

### Fractional atomic coordinates and isotropic or equivalent isotropic displacement parameters (Å^2^)

**Table:**

	*x*	*y*	*z*	*U*_iso_*/*U*_eq_	
C3	0.2646 (4)	0.9772 (3)	0.4633 (5)	0.0233 (13)	
H3A	0.2439	1.0130	0.5173	0.028*	
H3B	0.2335	0.9859	0.3748	0.028*	
C4	0.3620 (4)	0.9877 (3)	0.4750 (5)	0.0243 (13)	
H4A	0.3815	0.9562	0.4132	0.029*	
H4B	0.3734	1.0383	0.4551	0.029*	
C5	0.4128 (4)	0.9694 (3)	0.6083 (5)	0.0267 (13)	
H5A	0.4755	0.9727	0.6122	0.032*	
H5B	0.3985	1.0045	0.6691	0.032*	
C6	0.3905 (4)	0.8933 (3)	0.6443 (6)	0.0255 (14)	
H6A	0.4109	0.8580	0.5892	0.031*	
H6B	0.4210	0.8833	0.7326	0.031*	
C7	0.2938 (4)	0.8839 (3)	0.6313 (5)	0.0220 (13)	
H7A	0.2816	0.8332	0.6501	0.026*	
H7B	0.2746	0.9151	0.6937	0.026*	
C11	−0.0526 (3)	0.8256 (3)	0.5180 (4)	0.0175 (11)	
C12	−0.0838 (4)	0.8538 (3)	0.6181 (5)	0.0193 (11)	
H12	−0.0832	0.9044	0.6328	0.023*	
C13	−0.1159 (3)	0.8069 (3)	0.6957 (5)	0.0205 (12)	
H13	−0.1370	0.8248	0.7649	0.025*	
C14	−0.1167 (4)	0.7338 (3)	0.6710 (5)	0.0225 (12)	
C15	−0.0857 (4)	0.7057 (3)	0.5722 (5)	0.0266 (14)	
H15	−0.0870	0.6552	0.5571	0.032*	
C16	−0.0528 (4)	0.7522 (3)	0.4957 (5)	0.0218 (12)	
H16	−0.0303	0.7339	0.4280	0.026*	
Cl8	0.10439 (9)	0.97037 (6)	0.62798 (11)	0.0190 (3)	
Cl9	0.12310 (9)	0.80109 (7)	0.32660 (11)	0.0212 (3)	
N2	0.2441 (3)	0.9028 (2)	0.5016 (4)	0.0160 (9)	
H2	0.2629	0.8713	0.4469	0.019*	
N10	−0.0192 (3)	0.8739 (2)	0.4368 (4)	0.0157 (9)	
H10A	−0.0439	0.9185	0.4409	0.019*	
H10B	−0.0373	0.8576	0.3552	0.019*	
N17	−0.1522 (3)	0.6855 (3)	0.7523 (4)	0.0294 (12)	
O18	−0.1616 (3)	0.7083 (2)	0.8546 (3)	0.0316 (10)	
O19	−0.1721 (4)	0.6237 (2)	0.7150 (4)	0.0464 (14)	
Pt1	0.113556 (13)	0.887195 (10)	0.474086 (16)	0.01473 (9)	

### Atomic displacement parameters (Å^2^)

**Table:**

	*U*^11^	*U*^22^	*U*^33^	*U*^12^	*U*^13^	*U*^23^
C3	0.021 (3)	0.032 (3)	0.019 (3)	−0.008 (2)	0.007 (2)	0.001 (2)
C4	0.024 (4)	0.030 (3)	0.022 (3)	−0.003 (2)	0.010 (2)	0.000 (2)
C5	0.021 (3)	0.031 (3)	0.029 (3)	−0.001 (3)	0.007 (3)	−0.003 (3)
C6	0.022 (4)	0.026 (3)	0.028 (3)	0.000 (2)	0.005 (3)	0.000 (2)
C7	0.018 (3)	0.026 (3)	0.022 (3)	−0.001 (2)	0.003 (2)	0.003 (2)
C11	0.013 (3)	0.022 (3)	0.016 (2)	−0.002 (2)	0.000 (2)	0.000 (2)
C12	0.019 (3)	0.019 (3)	0.020 (3)	−0.001 (2)	0.006 (2)	−0.004 (2)
C13	0.013 (3)	0.034 (3)	0.014 (2)	−0.003 (2)	0.004 (2)	−0.002 (2)
C14	0.025 (3)	0.024 (3)	0.021 (3)	−0.004 (2)	0.010 (2)	0.005 (2)
C15	0.040 (4)	0.017 (3)	0.021 (3)	−0.001 (3)	0.004 (3)	−0.002 (2)
C16	0.025 (3)	0.021 (3)	0.020 (3)	−0.001 (2)	0.008 (2)	0.001 (2)
Cl8	0.0217 (8)	0.0195 (6)	0.0164 (6)	0.0017 (5)	0.0054 (5)	−0.0009 (5)
Cl9	0.0220 (8)	0.0239 (6)	0.0174 (6)	0.0023 (6)	0.0039 (5)	−0.0042 (5)
N2	0.012 (3)	0.020 (2)	0.016 (2)	−0.0003 (18)	0.0033 (18)	−0.0026 (18)
N10	0.013 (3)	0.019 (2)	0.015 (2)	0.0011 (18)	0.0022 (19)	−0.0009 (18)
N17	0.031 (3)	0.030 (3)	0.028 (3)	−0.003 (2)	0.009 (2)	0.006 (2)
O18	0.035 (3)	0.044 (2)	0.021 (2)	−0.007 (2)	0.0163 (18)	0.0022 (19)
O19	0.077 (4)	0.026 (2)	0.046 (3)	−0.006 (2)	0.034 (3)	0.003 (2)
Pt1	0.01469 (15)	0.01670 (13)	0.01350 (12)	0.00081 (7)	0.00464 (9)	0.00004 (7)

### Geometric parameters (Å, º)

**Table:**

C3—H3A	0.9900	C12—H12	0.9500
C3—H3B	0.9900	C12—C13	1.387 (7)
C3—C4	1.535 (8)	C13—H13	0.9500
C3—N2	1.497 (6)	C13—C14	1.381 (7)
C4—H4A	0.9900	C14—C15	1.382 (7)
C4—H4B	0.9900	C14—N17	1.458 (7)
C4—C5	1.528 (8)	C15—H15	0.9500
C5—H5A	0.9900	C15—C16	1.380 (7)
C5—H5B	0.9900	C16—H16	0.9500
C5—C6	1.526 (7)	Cl8—Pt1	2.3039 (11)
C6—H6A	0.9900	Cl9—Pt1	2.2917 (12)
C6—H6B	0.9900	N2—H2	0.9300
C6—C7	1.520 (8)	N2—Pt1	2.046 (4)
C7—H7A	0.9900	N10—H10A	0.9200
C7—H7B	0.9900	N10—H10B	0.9200
C7—N2	1.492 (7)	N10—Pt1	2.068 (4)
C11—C12	1.396 (7)	N17—O18	1.231 (5)
C11—C16	1.382 (7)	N17—O19	1.232 (6)
C11—N10	1.440 (6)		
			
H3A—C3—H3B	107.9	C13—C12—H12	120.5
C4—C3—H3A	109.3	C12—C13—H13	120.5
C4—C3—H3B	109.3	C14—C13—C12	119.0 (5)
N2—C3—H3A	109.3	C14—C13—H13	120.5
N2—C3—H3B	109.3	C13—C14—C15	122.2 (5)
N2—C3—C4	111.8 (4)	C13—C14—N17	118.2 (5)
C3—C4—H4A	109.5	C15—C14—N17	119.6 (5)
C3—C4—H4B	109.5	C14—C15—H15	120.6
H4A—C4—H4B	108.1	C16—C15—C14	118.9 (5)
C5—C4—C3	110.8 (4)	C16—C15—H15	120.6
C5—C4—H4A	109.5	C11—C16—H16	120.1
C5—C4—H4B	109.5	C15—C16—C11	119.7 (5)
C4—C5—H5A	109.6	C15—C16—H16	120.1
C4—C5—H5B	109.6	C3—N2—H2	106.2
H5A—C5—H5B	108.1	C3—N2—Pt1	111.5 (3)
C6—C5—C4	110.2 (5)	C7—N2—C3	112.2 (4)
C6—C5—H5A	109.6	C7—N2—H2	106.2
C6—C5—H5B	109.6	C7—N2—Pt1	113.9 (3)
C5—C6—H6A	109.3	Pt1—N2—H2	106.2
C5—C6—H6B	109.3	C11—N10—H10A	108.0
H6A—C6—H6B	107.9	C11—N10—H10B	108.0
C7—C6—C5	111.7 (5)	C11—N10—Pt1	117.0 (3)
C7—C6—H6A	109.3	H10A—N10—H10B	107.3
C7—C6—H6B	109.3	Pt1—N10—H10A	108.0
C6—C7—H7A	109.3	Pt1—N10—H10B	108.0
C6—C7—H7B	109.3	O18—N17—C14	118.7 (4)
H7A—C7—H7B	108.0	O18—N17—O19	122.8 (5)
N2—C7—C6	111.5 (4)	O19—N17—C14	118.5 (5)
N2—C7—H7A	109.3	Cl9—Pt1—Cl8	177.84 (4)
N2—C7—H7B	109.3	N2—Pt1—Cl8	91.59 (12)
C12—C11—N10	119.4 (4)	N2—Pt1—Cl9	88.59 (12)
C16—C11—C12	121.2 (5)	N2—Pt1—N10	176.94 (15)
C16—C11—N10	119.4 (4)	N10—Pt1—Cl8	89.56 (12)
C11—C12—H12	120.5	N10—Pt1—Cl9	90.37 (12)
C13—C12—C11	119.0 (5)		
			
C3—C4—C5—C6	54.8 (6)	C12—C11—N10—Pt1	97.9 (5)
C3—N2—Pt1—Cl8	−66.5 (3)	C12—C13—C14—C15	0.7 (9)
C3—N2—Pt1—Cl9	115.7 (3)	C12—C13—C14—N17	−178.9 (5)
C4—C3—N2—C7	54.8 (6)	C13—C14—C15—C16	0.0 (9)
C4—C3—N2—Pt1	−176.0 (3)	C13—C14—N17—O18	−16.4 (8)
C4—C5—C6—C7	−55.5 (6)	C13—C14—N17—O19	163.1 (6)
C5—C6—C7—N2	55.4 (6)	C14—C15—C16—C11	−0.9 (8)
C6—C7—N2—C3	−54.8 (6)	C15—C14—N17—O18	164.0 (5)
C6—C7—N2—Pt1	177.4 (3)	C15—C14—N17—O19	−16.5 (8)
C7—N2—Pt1—Cl8	61.7 (3)	C16—C11—C12—C13	−0.3 (8)
C7—N2—Pt1—Cl9	−116.1 (3)	C16—C11—N10—Pt1	−81.8 (5)
C11—C12—C13—C14	−0.6 (8)	N2—C3—C4—C5	−54.9 (6)
C11—N10—Pt1—Cl8	−82.4 (3)	N10—C11—C12—C13	180.0 (5)
C11—N10—Pt1—Cl9	95.5 (3)	N10—C11—C16—C15	−179.2 (5)
C12—C11—C16—C15	1.1 (8)	N17—C14—C15—C16	179.6 (5)

### Hydrogen-bond geometry (Å, º)

**Table:**

*D*—H···*A*	*D*—H	H···*A*	*D*···*A*	*D*—H···*A*
N2—H2···O18^i^	0.93	2.27	3.182 (6)	165
N10—H10*A*···Cl8^ii^	0.92	2.32	3.198 (4)	158
N10—H10*B*···Cl9^iii^	0.92	2.37	3.255 (4)	161

Symmetry codes: (i) *x*+1/2, −*y*+3/2, *z*−1/2; (ii) −*x*, −*y*+2, −*z*+1; (iii) −*x*, *y*, −*z*+1/2.
